# The role of receptor MAS in microglia-driven retinal vascular development

**DOI:** 10.1007/s10456-019-09671-3

**Published:** 2019-06-20

**Authors:** S. Foulquier, V. Caolo, G. Swennen, I. Milanova, S. Reinhold, C. Recarti, N. Alenina, M. Bader, U. M. Steckelings, T. Vanmierlo, M. J. Post, E. A. Jones, R. J. van Oostenbrugge, T. Unger

**Affiliations:** 1 Department of Pharmacology-Toxicology, Maastricht University, PO Box 616, 6200 MD Maastricht, The Netherlands; 2 Department of Cardiovascular Sciences, Centre for Molecular and Vascular Biology, KU Leuven, Leuven, Belgium; 3 Department of Physiology, Maastricht University, Maastricht, The Netherlands; 4 Department of Molecular Cell Biology, Maastricht University, Maastricht, The Netherlands; 5 Max Delbruck Center for Molecular Medicine, Berlin, Germany; 6 Partner Site Berlin, DZHK (German Center for Cardiovascular Research), Berlin, Germany; 7 Berlin Institute of Health (BIH), Berlin, Germany; 8 Charité - University Medicine, Berlin, Germany; 9 Institute for Biology, University of Lübeck, Lübeck, Germany; 10 Institute of Molecular Medicine, Department of Cardiovascular & Renal Research, University of Southern Denmark, Odense, Denmark; 11 Department of Immunology and Biochemistry, Biomed, Hasselt University, Diepenbeek, Belgium; 12 Department of Neurology, Maastricht University Medical Center, Maastricht, The Netherlands; 13 Cardiovascular Research Institute Maastricht, CARIM, Maastricht, The Netherlands; 14 MH&NS, School for Mental Health and Neuroscience, Maastricht University, Maastricht, The Netherlands

**Keywords:** Angiogenesis, Renin angiotensin system, Angiotensin receptors, Macrophage, CNS, Developmental biology, Endothelium, Vascular biology

## Abstract

**Objective:**

The receptor MAS, encoded by *Mas1*, is expressed in microglia and its activation has been linked to anti-inflammatory actions. However, microglia are involved in several different processes in the central nervous system, including the promotion of angiogenesis. We therefore hypothesized that the receptor MAS also plays a role in angiogenesis via microglia.

**Approach and results:**

To assess the role of MAS on vascular network development, flat-mounted retinas from 3-day-old wild-type (WT) and Mas1^−/−^ mice were subjected to Isolectin B4 staining. The progression of the vascular front was reduced (− 24%, *p* < 0.0001) and vascular density decreased (− 38%, *p* < 0.001) in Mas1^−/−^ compared to WT mice with no change in the junction density. The number of filopodia and filopodia bursts were decreased in Mas1^−/−^ mice at the vascular front (− 21%, *p* < 0.05; − 29%, *p* < 0.0001, respectively). This was associated with a decreased number of vascular loops and decreased microglial density at the vascular front in Mas1^−/−^ mice (-32%, *p* < 0.001; − 26%, *p* < 0.05, respectively). As the front of the developing vasculature is characterized by reduced oxygen levels, we determined the expression of *Mas1* following hypoxia in primary microglia from 3-day-old WT mice. Hypoxia induced a 14-fold increase of *Mas1* mRNA expression (*p* < 0.01). Moreover, stimulation of primary microglia with a MAS agonist induced expression of *Notch1* (+ 57%, *p* < 0.05), *Dll4* (+ 220%, *p*  < 0.001) and *Jag1* (+ 137%, *p* < 0.001), genes previously described to mediate microglia/endothelial cell interaction during angiogenesis.

**Conclusions:**

Our study demonstrates that the activation of MAS is important for microglia recruitment and vascular growth in the developing retina.

**Electronic supplementary material:**

The online version of this article (10.1007/s10456-019-09671-3) contains supplementary material, which is available to authorized users.

## Introduction

The development of the central nervous system (CNS) vasculature is a complex process driven by numerous signalling pathways and the interaction of different cell types. Beyond the contribution of mural cells that provide support and guidance to nascent vessels, microglia, the resident immune cells of the CNS, appear to play a key role in shaping the structure of the developing vasculature.

Microglia have been extensively studied in the context of brain injury and for their immune function as guardians of the CNS [1–3]; however, they appear to perform many other tasks, even in their “resting” state, such as monitoring neuronal activity, pruning, maturation and elimination of synapses as well as blood vessel formation [4, 5]. Microglia role in vascular network formation has been corroborated by studies showing their involvement in the onset of vasculogenesis in human foetal retina at 10 weeks of gestation before the development of the retinal vasculature and in growing sprouting vessels [6]. Furthermore, retinal microglia depletion was associated with a reduced vascular development [4]. Throughout the developing retina, including the tip and stalk cells of the vascular front, all endothelial cells appeared to be in intimate proximity of microglia [4]. Although cultured microglia are known to release growth factors and matrix metalloproteinases (MMP) of importance for endothelial cell proliferation and migration (e.g. VEGF, bFGF, MMP9 [7–11]), more studies are required to decipher the exact molecular interactions between microglia and endothelial cells during vascular development.

The Renin Angiotensin System (RAS) is one of the major endocrine systems involved in the regulation of blood pressure and fluid homeostasis. In addition, the RAS plays a major role during organogenesis, especially of the kidney and heart [12–14]. The RAS is also expressed in developing retinas in rats and in humans [15, 16]. Over the last decade, new RAS components have been identified. The ACE2 enzyme, a homolog of ACE, can cleave Ang II into Ang-(1–7). By binding to its receptor MAS (encoded by the *Mas1* gene), Ang-(1–7) mediates vasodilation, anti-proliferation, anti-fibrosis, apoptosis and anti-inflammation [17]. For these reasons, the ACE2/Ang-(1–7)/Mas axis is considered a so-called “protective arm” of the RAS, in opposition to the actions mediated by the ACE/Ang II/AT_1_ axis [17, 18].

While previous studies have reported the anti-inflammatory actions induced by activation of the receptor MAS in microglia [19], its role in angiogenesis remains controversial. Although many studies centred on tumorigenesis have reported an anti-angiogenic role for the Ang-(1–7)/Mas axis [20–27], a few studies found that MAS promotes angiogenesis [28, 29]. Hoffman et al. recently showed that low doses of Ang-(1–7) induce tube formation of rat microvascular endothelial cells via MAS stimulation and subsequent activation of ERK1/2 [30]. In particular, in the CNS, *Mas1* is expressed in both endothelial and microglia cells [31]. The high expression level of *Mas1* during development suggests that it must be of importance in developmental stages but its role in neonatal vascularization has never been studied [31].

Here we show that neonatal retina from *Mas1*-deficient (Mas1^−/−^ ) mice has impaired vascular development and this is linked to reduced number of microglia at the front of the developing retinal vasculature. Both progression of vascular front and filopodia sprouting were decreased in retinas from Mas1^−/−^ as compared to wild-type (WT) mice. Remarkably, the number of microglia at the vascular front was also decreased, whereas it did not change in other regions of the retinas. The region, in which the filopodia burst and the vascular front progresses, is highly hypoxic. We found that *Mas1* is strongly upregulated in microglia in hypoxic conditions. Stimulation of the receptor MAS with the non-peptide Ang-(1–7) analogue, AVE0991, resulted in the upregulation of *Notch1*, Delta-like ligand 4 (*Dll4*) and Jagged1 (*Jag1*) expression, members of Notch signalling pathway previously described to play a role in microglia recruitment at the sites of endothelial cell anastomosis during retinal angiogenesis [32]. Altogether, these results indicate a role for MAS in microglia/endothelial cell interaction for the growth of developing vascular network in the neonatal mouse retina.

## Material and methods

### Animals

Three-day-old *Mas1*-deficient (Mas1^−/−^) mice and C57BL/6J wild-type (WT) mice were obtained from the Max Delbrück Center for Molecular Medicine, Berlin, Germany [33] and were sacrificed at postnatal day 3 for the study of retinas. For the isolation of primary microglia, 2–3-day-old C57BL/6J WT mice were obtained from BIOMED Research Institute, Hasselt University, Belgium. Animals were maintained under temperature-controlled conditions with an artificial 12-h light/dark cycle and were allowed standard chow and water ad libitum. All experimental protocols and methods involving animals within this study were conducted in accordance with institutional guidelines and approved by the Ethical Committees for Animal Experiments from Max Delbrück Center for Molecular Medicine, Berlin, and Hasselt University.

### Retina isolation and immunohistochemistry

Retinas were isolated from the eyeballs of 3-day-old WT and Mas1^−/−^ mice as previously described (*n* = 8) [34]. Isolectin B4 (IB4, from *Griffonia simplicifolia*, Alexa Fluor® 488 conjugate, I21411, Thermo Fisher Scientific, 20 µg/ml) was used to stain blood vessels [35]. Microglia were stained with a rabbit polyclonal anti-Iba1 antibody (Ionized calcium-Binding Adapter molecule 1, a specific microglia marker) (Wako 019-19741, 1:500) and secondary goat anti-rabbit AF568 antibody (Invitrogen A11036, 1:400). Retinas were flat-mounted with a drop of ProLong® Diamond Antifade Mountant (Thermo Fisher P36965). The vascular front was measured as the radius of the vascular network from the centre of the optic disc to the edge of the network, using low-magnification (× 2) pictures acquired with an EVOS-FL microscope. To quantify the other vascular parameters, pictures were taken with a confocal Leica SPE microscope. Vascular density (calculated as percentage of the field of view stained by IB4) and junctions densities (calculated as the number of vessel junctions per mm^2^) were obtained using ImageJ and Angiotool software [36], using 4–6 fields of views away from the vascular front, per retina at × 10 and × 20 magnification, respectively. Filopodia density, filopodia burst densities, the number of sprouts, the average vascular sprout length and the number of vascular loops (defined as anastomosis of vascular sprouts) at the vascular front were analysed from eight fields of views from 6 to 9 retinas acquired at a × 63 magnification (*x* 175; *y* 175; *z* 18 µm) [37]. Number of filopodia and number of sprouts were quantified per 100 µm vascular front length and number of filopodia bursts and number of vascular loops were quantified per 0.03 mm^2^. Density of microglia was assessed in both avascular and vascular areas of WT and Mas1^−/−^ retinas (*n* = 12–14). Four z-stacks were acquired per retina (*x* 300; *y* 300; *z* 18 µm) and were maximally projected to quantify the number of Iba1 positive cells per area.

### Microglia isolation, immunocytochemistry, stimulation

Microglia cultures were prepared from postnatal P2-3 C57BL/6J mouse pups and cultured as previously described [38]. Purity of microglial culture was assessed by IB4/DAPI staining and revealed 91.0 ± 0.6% IB4^+^ cells (data not shown). Microglia were stained for Iba1 with a rabbit polyclonal anti-Iba1 antibody (Wako 019-19741, 1:500) and secondary goat anti-rabbit AF568 antibody (Invitrogen A11036, 1/400). The expression of the receptor MAS was revealed using an anti-MAS antibody (AAR-013, Alomone Labs, 1:20) and a secondary goat anti-rabbit antibody coupled to Alexa Fluor 568 (Invitrogen, A11036, 1:200) (Supplementary Fig. 1). Prior to RNA collection, microglia were exposed to the non-peptide MAS agonist (AVE 0991, ApexBio B1007, 1 µM, 24 h) or to hypoxia to mimic the absence of vasculature (1% O_2_, 24 h).

### Tube formation assay

Geltrex™ LDEV-Free Reduced Growth Factor Basement Membrane Matrix (ThermoFisher, A1413201) was used for tube formation assays with RF6/A cells (ATCC CRL-1780) in 96-well microplate (µ-Plate Angiogenesis 96 Well, ibidi®, 89646) by following the manufacturer’s instructions. RF/6A cells are spontaneously immortalized endothelial cells derived from the choroid and retina of a rhesus macaque. RF/6A cells were cultured at 37 °C, in 5% CO_2_ and 95% humidified air in EMEM (ATCC 30-2003) supplemented with 10% foetal bovine serum (FBS, Gibco) and 1% penicillin/streptomycin (Gibco). Before the cells reach confluency, they were trypsinized, resuspended in EMEM + 0.5% FBS with the different conditions (PBS, VEGF 1 nM as positive control, Suramin 10 µM, *an inhibitor of several growth factor receptors*, as negative control or AVE0991 1 µM, non-peptide MAS agonist) before seeding on Geltrex-coated wells (10 000 cells/well). At *t* = 6 h, cells were incubated with CellTracker™ Green CMFDA (Thermo Fisher Scientific C7025, 15 µM) for 30 min before washing with PBS and imaging. Wells were imaged with Cytation 3 microplate reader (BioTek Instruments Inc.) using a × 4 objective and a GFP filter and tube formation analysis was performed with the AngioTool software [36].

### Quantitative PCR

RNA was isolated from cells by using RNeasy micro kit Qiagen (Qiagen, GmbH, Hilden, Germany). DNA was synthesized with Applied Biosystems High-Capacity cDNA Reverse Transcription Kit. Quantitative real-time PCR was performed on a Biorad CFX96 (Biorad, Veenendaal, The Netherlands). Primers are listed in Supplementary Table 1. The level of expression was normalized and expressed relatively to control using the ΔΔCt method. Two experiments were performed per condition and PCR reactions were performed in triplicate.

### Statistical analysis

Statistical analyses were performed using GraphPad Prism v6.0. Results are expressed as mean ± SEM. Unpaired two-tailed *t*-tests were used to compare results from WT and Mas1^−/−^ groups. One-way ANOVA, followed by Tukey’s multiple comparison post-test was used to compare results from the tube formation assay experiments.

## Results

### *Mas1* deficiency impairs retinal vasculature development

**Fig. 1:**
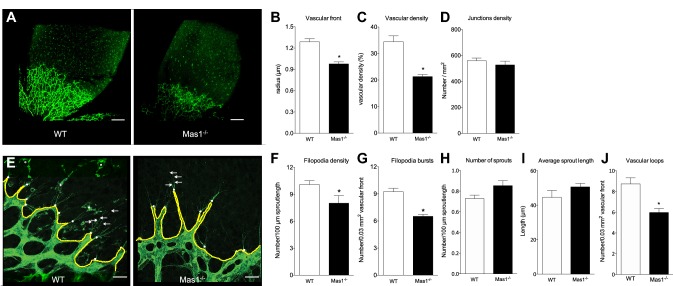
Retinal vasculature in 3-day-old WT and Mas1^−/−^ mice (*n* = 8). Representative image of retinal vasculature stained with IB4 (green) (**a**, scale bar = 300 µm); Vascular front (**b**), vascular density (**c**) and junctions density (**d**); (*n* = 6–8). Representative pictures of vascular sprouts and filopodia at the vascular front (**e**; arrows: filopodia; yellow line: vascular front; white dots: filopodia bursts; stars: microglia; scale bars = 20 µm); Filopodia density (**f**), filopodia burst densities (**g**), number of sprouts (**h**), average sprout length (**i**) and number of vascular loops (**j**) at the vascular front of retinas from WT and Mas1^−/−^ mice. *p < 0.05 versus WT

In order to study the role of MAS in developmental angiogenesis, we performed Isolectin B4 (IB4) staining of retinal vasculature from 3-day-old Mas1^−/−^ mice (*n* = 8). We found that MAS deficiency was associated with decreased progression of the vascular front (− 24%, *p* < 0.0001) (Fig. 1a, b) and decreased vascular density (− 38%, *p* < 0.001) (Fig. 1c) without alteration of junction density (− 6%, *p* = 0.37) (Fig. 1d). Moreover, the densities of filopodia and filopodia bursts were decreased in absence of MAS (− 21%, *p* < 0.05; and − 29%, *p* < 0.0001, respectively) (Fig. 1e–g). There was no difference between groups in the average length of sprouts or the number of sprouts (+ 14%, *p* = 0.16 and + 17%, *p* = 0.05, respectively) (Fig. 1h, i). The number of vascular loops at the vascular front was decreased in Mas1^−/−^ (− 32%, *p* < 0,001) (Fig. 1j).

### MAS is important for microglia recruitment at the vascular front

**Fig. 2:**
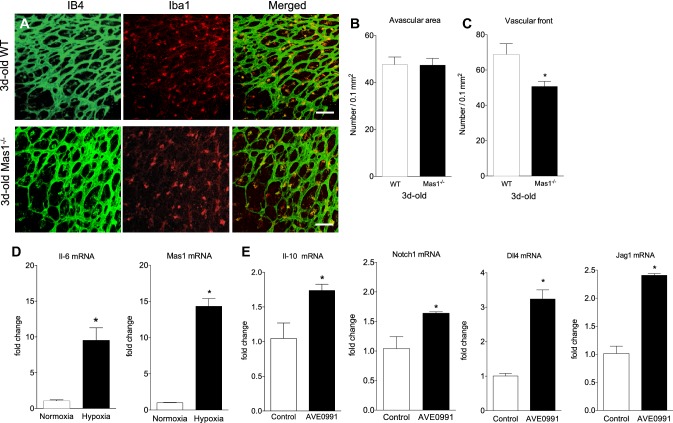
Retinal microglial densities. Vasculature (IB4) and microglia (Iba1) at the vascular front of retinas from 3-day-old WT and Mas1^−/−^ mice (**a**); microglial density in an avascular area (**b**) and at the vascular front (**c**); (*n* = 5–9). *Il-6* and *Mas1* mRNA expressions by primary microglia during hypoxia (**d**); *Il-10, Notch1, Dll4* and *Jag1* mRNA expressions in microglia treated with a MAS agonist (AVE0991, 1 µM) (**e**); (*n* = 2 experiments, triplicates). *p < 0.05 versus WT

The reduced growth of the vascular network was associated with a decreased microglial density at the vascular front of Mas1^−/−^ mice (− 26%, *p* < 0.05), while microglial density in the avascular area remained unchanged (*p* = 0.96) (Fig. 2a–c). Isolated microglia were positively stained by IB4 and expressed Iba1 and MAS (Supplementary Fig. 1). Microglia cells reacted to hypoxia by upregulating *Il-6* mRNA expression (eightfold change, *p* < 0.01) as well as *Mas1* mRNA expression (14-fold change, *p* < 0.01) (Fig. 2d). Upon MAS stimulation with AVE0991, *Il-10, Notch1, Dll4*, and *Jag1* mRNA expressions were upregulated (+ 67%, *p* < 0.05; + 57%, *p* < 0.05; + 220%, *p* < 0.001; + 137%, *p* < 0.001; respectively) (Fig. 2e).

### MAS stimulation promotes the formation of endothelial tubes in vitro

**Fig. 3:**
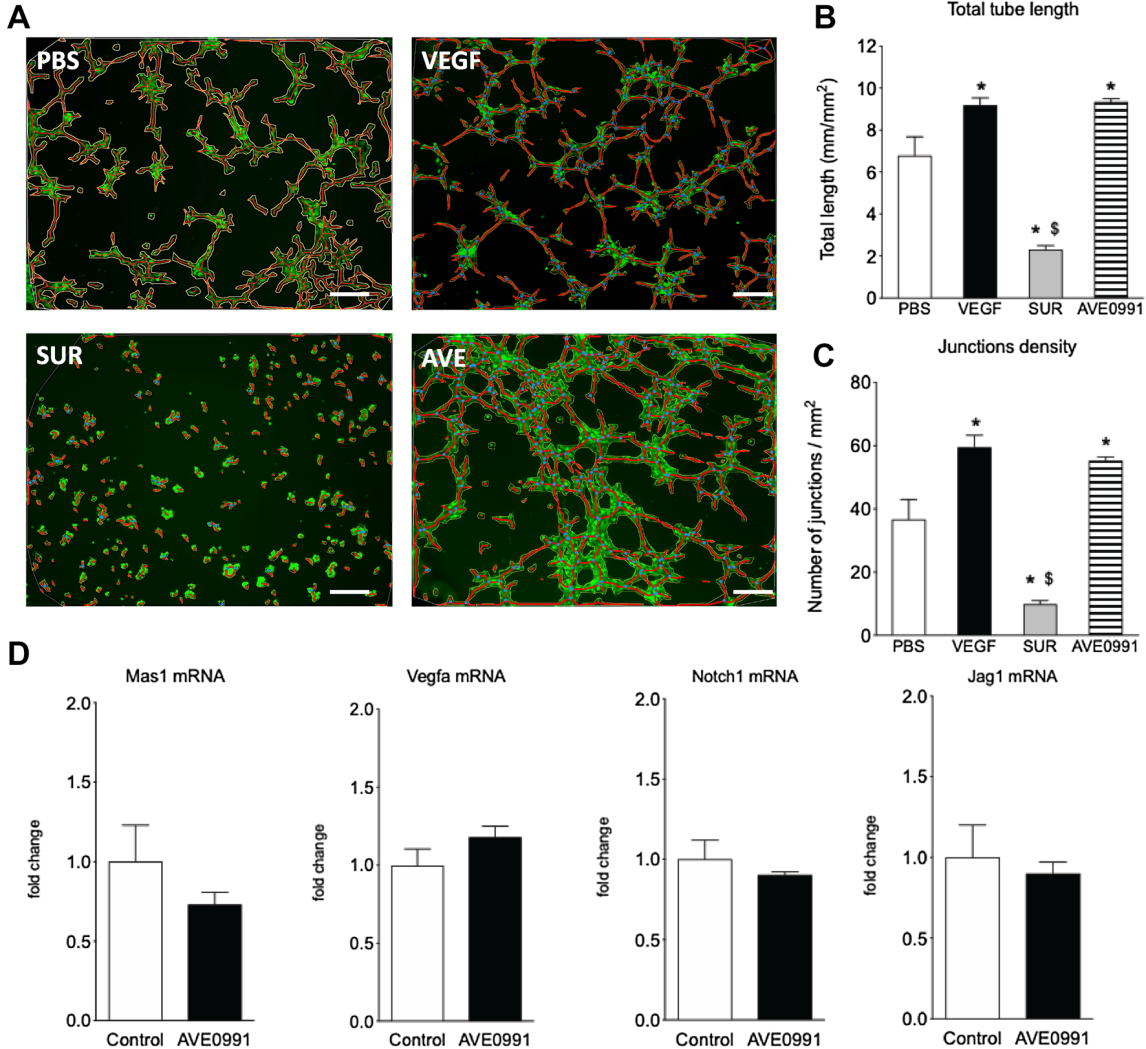
Impact of MAS stimulation on retinal endothelial cells in vitro. Tube formation assay with RF/6A cells (**a**–**c**) treated with PBS, VEGF (1 nM), Suramin (SUR, 10 µM) or a MAS receptor agonist (AVE0991, 1 µM). Representative pictures (**a**, scale bar = 200 µm); Quantification of total tube length (**b**) and junctions density (**c**) (*n* = 6–8); One-way ANOVA, followed by Tukey’s multiple comparisons post-test (*p < 0.05 vs. PBS; ^$^*p* < 0.05 vs. VEGF). *Mas1, Vegfa, Notch1* and *Jag1* mRNA expressions in RF/6A cells treated with a MAS agonist (AVE0991, 1 µM) (**d**); (*n* = 2 experiments, triplicates)

The formation of tubes (capillary-like structures) by retinal endothelial cells was enhanced by VEGF and the MAS agonist AVE0991 and abolished by Suramin treatment (Fig. 3a). Both the total tube length and number of junctions were increased by VEGF (+ 35%, *p* < 0.05; + 64%, *p* < 0.01, respectively) and by AVE0991 (+ 37%, *p* < 0.05; + 50%, *p* < 0.05, respectively)(Fig. 3b, c). Differently from what observed in microglia, stimulation of endothelial cells with AVE0991 did not affect *Mas1, Vegfa, Notch1, Jag1* mRNA expressions (Fig. 3d).

## Discussion

**Fig. 4:**
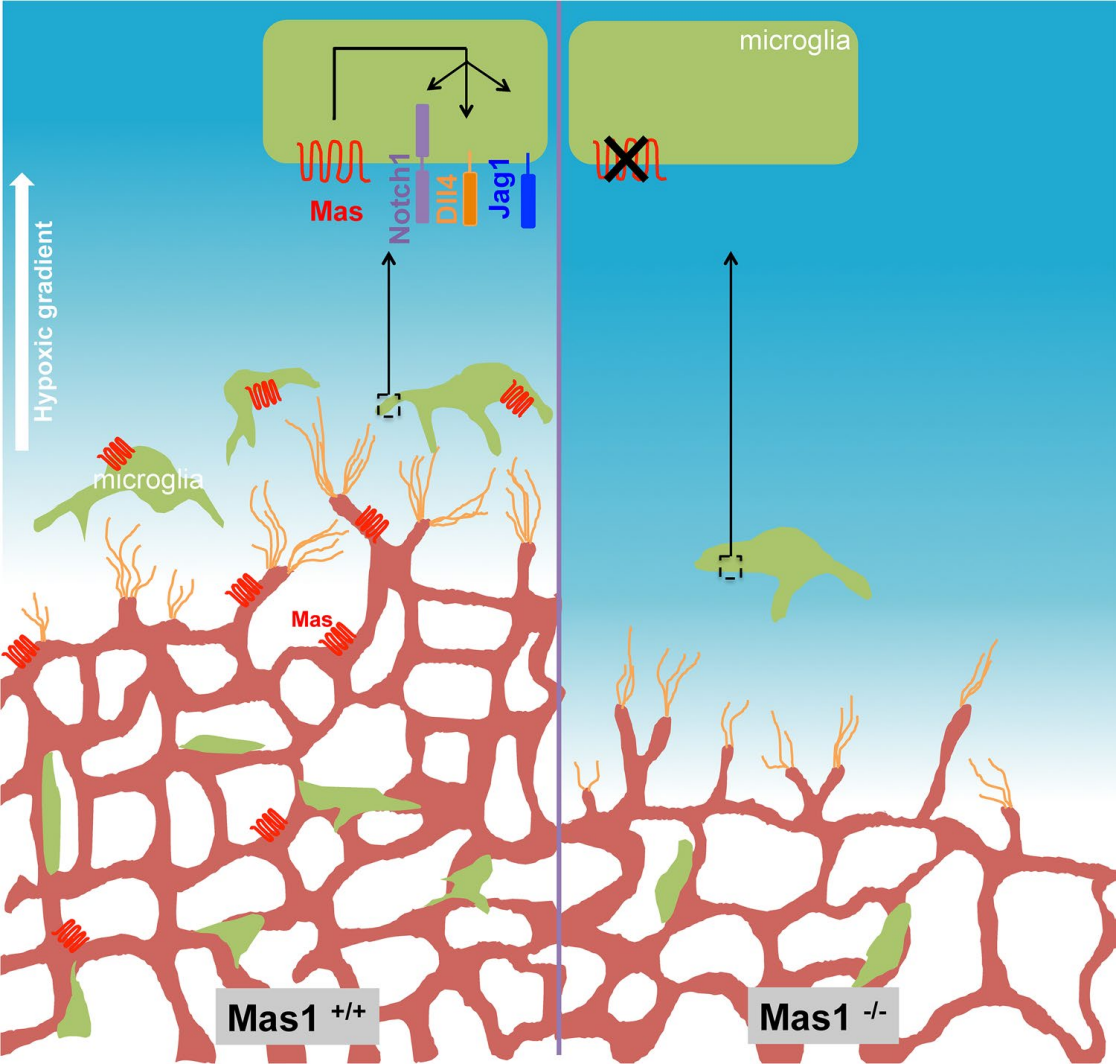
Schematic representation of the importance of *Mas1* in retinal vascular development

In this study, we show that MAS is required for microglia recruitment and blood vessel growth at the vascular front of developing retinas. The relative low oxygen concentration at the vascular front is a known driving force for the formation of new blood vessels in the developing retina [39]. The close positioning of microglia near endothelial tip cells and their exposure to low oxygen concentration has been shown to be of importance for the promotion of vascular sprouting under physiologic condition [4, 35]. In our study, hypoxic microglia upregulated the expression of *Mas1*, suggesting its importance for the function of microglia at the vascular front. In the absence of MAS, the number of filopodia and filopodia bursts were decreased, leading to a shorter vascular front and overall decreased vascular density. At the same time, the number of microglia at the vascular front was decreased in Mas1^−/−^ mouse indicating a possible role for MAS in the migration of microglia towards the vascular front (Fig. 4).

Noteworthy, we found that stimulation of microglia with the MAS agonist AVE0991 induces the expression of *Notch1* and its ligands *Dll4* and *Jag1*. Notch signalling pathway has been reported to be important for microglia recruitment at the vascular front in the neonatal developing retina [32], where microglia interact with sprouting endothelial cells to facilitate anastomosis between adjacent cells [4, 40]. Outtz et al. showed that recruitment of myeloid cells at the vascular front of Notch1^+/−^ mice was altered when compared to control [32]. Also, loss of Notch1 in myeloid lineage showed increased frequency of elongated tip endothelial cell sprout that failed to form anastomoses with their adjacent sprouts in the developing retinal vasculature [32]. In our study, the number of vascular loops at the vascular front was reduced in Mas1^−/−^ compared to WT and was associated with no change in number of sprouts in Mas1^−/−^, corroborating the findings from Outtz et al.

Besides *Notch1* upregulation, stimulation of microglia with AVE0991 resulted in the increased expressions of genes coding for Notch ligands, *Dll4* and *Jag1*. The understanding of *Dll4* and *Jag1* roles in microglia is limited. There are evidences pointing out a role for Notch1, Dll4 and Jag1 in macrophages and/or microglia in the context of inflammation. While *Dll4* seems involved in macrophage differentiation [41], *Notch1* and *Jag1* expressions were increased in activated microglia similarly to our study. Furthermore, antibody-mediated depletion of *Notch1* in microglia decreased the expression of pro-inflammatory cytokines as well as the expression of colony stimulating factor 1 (CSF1), a growth factor required for the proliferation, differentiation and survival of macrophages and microglia [42]. This latter finding, together with the increased *Notch1* expression mediated by MAS stimulation in our study, lets us suggest that the decreased microglial density at the vascular front may also result from a decreased release of CSF1 by microglia in Mas1^−/−^ mice.

In addition, endothelial tip cells have been described to have low Notch signal, whereas their neighbour stalk cells show high Notch signal. However, tip cells continuously swap their positions, allowing stalk cells to become tip cells and *vice versa* [43, 44]. Therefore, it is reasonable to speculate that microglial Jag1 would activate Notch in the tip cells, providing a mechanism for the tip cells to become stalk cells, allowing other endothelial tip cells to take the lead for the progression of sprouting process and vascular network growth.

Upon exposure to hypoxia, microglia acquired a pro-inflammatory phenotype with an increased *Il-6* expression as expected [45], whereas the activation of the receptor MAS led to the upregulation of the anti-inflammatory cytokine *Il-10*, as previously demonstrated [19]. The anti-inflammatory and pro-angiogenic effect associated with MAS signalling may constitute a key mechanism to protect the CNS from injuries. In fact, it has been shown that the stimulation of the Ang-(1–7)/Mas axis is cerebroprotective in the context of stroke by increasing the brain capillary density via the activation of an eNOS/NO/VEGF signalling [29]. In diabetic animal models, a recent study has shown an imbalance between Ang II and Ang-(1–7) in the diabetic eyes [45]. While in the non-diabetic eyes, Ang-(1–7) has a wider distribution than Ang II, its intensity and distribution were both reduced in diabetic animals [45]. This study showed additionally that the pharmacological inhibition of ACE with captopril could reverse this imbalance and suggests that increasing the endogenous expression of Ang-(1–7) could be beneficial in the context of diabetic retinopathy [46]. Another recent study has shown that low doses of Ang-(1–7) induce tube formation of rat microvascular endothelial cells [30]. Similarly, we found that stimulation of endothelial cells with the MAS agonist AVE0991 induces retinal endothelial cells to form tubes and this was observed in the absence of the regulation of *Mas1, Vegfa, Notch1* and *Jag1*. Of note, while the Ang-(1–7)/Mas axis has been often described for having similar functions than the Ang II/ Ang II receptor type 2 (AT_2_) axis, the second key player of the protective RAS [47], we did not observe changes in the retinal vasculature of AT_2_^−/−^ mice (data not shown).

We found that MAS stimulation induces expression of Notch signalling members in microglia, but not endothelial cells. We can however not draw conclusions on the impact of hypoxia on Notch signalling in the absence of MAS. Deeper insights into the interaction between MAS and Notch signalling pathways could provide new and more effective therapeutic strategies to contain pathological NOTCH-stimulated angiogenesis, in diseases such as cancer or diabetic retinopathy.

## Electronic supplementary material

Below is the link to the electronic supplementary material.

Supplementary material 1 (DOCX 40301 kb)
