# Supplementary material for: The role of receptor MAS in microglia-driven retinal vascular development
Source: Angiogenesis. 2019 Jun 20;22(4):481–9. doi: 10.1007/s10456-019-09671-3 (PMC6863789; doi:10.1007/s10456-019-09671-3)
Supplement: Supplementary file 1 — Supplementary material 1 (DOCX 40301 kb) [file 10456_2019_9671_MOESM1_ESM.docx]

Supplementary Table 1: Primers list

| Mouse primers | |
| --- | --- |
| Il-6 | Taqman Gene expression assay Mm00446190_m1 |
| Il-10 | Taqman Gene expression assay Mm00439614_m1 |
| Mas1 | Taqman Gene expression assay Mm00434823_s1 |
| Gapdh | Taqman Gene expression assay Mm99999915_g1 |
| Vegfa | Fw GTCAGAGAGCAACATCACCA; Rev CATCTGCTGTGCTGTAGGAA |
| Notch1 | Fw AGCCTCTCCACCAATACCTT; Rev GGCTGGAGCTGTAAGTTCTG |
| Dll4 | Fw ACTTCGTCTGCAACTGTCCT; Rev CAGCACCAGCAGTACCACTA |
| Jag1 | Fw CCTCTGCTGAGCTCTGTCTT; Rev ATTGTTGGTGGTGTTGTCCT |
| Gapdh | Fw AAGTGGAGATTGTTGCCATC; Rev CGTGAGTGGAGTCATACTGG |
| Macaca mulatta primers | |
| ACTB | Fw AAGATCAAGATCATTGCTCCTCCT; Rev GATGGAAGGGCCAGACTCG |
| ALAS1 | Fw TCAATGGATGGGGCAGTGTG; Rev TCCACGAAGGTGATTGCTCC |
| MAS1 | Fw GCCTGTCAGTCCTTTACCCC; Rev ACAGAAGGGCACAGACCAAC |
| VEGFA | Fw CACGAAGTGGTGAAGTTCATGG; Rev GGCACACAGGATGGCTTGAA |
| NOTCH1 | Fw GACTCAGCAGCACCTGGATG; Rev GGGGTGAAGCCGTCAGG |
| JAG1 | Fw TTGCCAGCTACTACTGCGAC; Rev AACCAAATCCCGACAGGAGG |

Supplementary Figure 1: Representative pictures of isolated microglia co-stained with DAPI. Microglia were positively stained by IsolectinB4 (IB4) and expressed Iba1 and the receptor MAS (Scale bars = 50 µm).
